# Right Ventricular Dysfunction Staging System for Mortality Risk Stratification in Heart Failure with Preserved Ejection Fraction

**DOI:** 10.3390/jcm9030831

**Published:** 2020-03-18

**Authors:** Enrique Santas, Rafael De la Espriella, Francisco Javier Chorro, Patricia Palau, Gema Miñana, Raquel Heredia, Martina Amiguet, Héctor Merenciano, Juan Sanchis, Josep Lupón, Antoni Bayés-Genís, Julio Núñez

**Affiliations:** 1 Cardiology Department, Hospital Clínico Universitario, Universitat de València, INCLIVA, CIBERCV, avenida Blasco Ibáñez 17, 46010 Valencia, Spain; ensantas@gmail.com (E.S.); rdelaespriella@gmail.com (R.D.l.E.); Francisco.J.Chorro@uv.es (F.J.C.); gemineta@gmail.com (G.M.); rhcambra@gmail.com (R.H.); martinna1990@gmail.com (M.A.); hectormeren@gmail.com (H.M.); sanchis_juafor@gva.es (J.S.); 2 Cardiology Department, Hospital General de Castellón, Universitat Jaume I, avenida de Benicassim 128, 12004 Castellón, Spain; patripalau@gmail.com; 3 Cardiology Department, Hospital Germans Trias i Pujol, Universitat Autònoma de Barcelona, CIBERCV, carretera de Canyet s/n, 08196 Badalona, Spain; jlupon.germanstrias@gencat.cat (J.L.); abayesgenis@gmail.com (A.B.-G.)

**Keywords:** heart failure with preserved ejection fraction, right ventricular, risk stratification

## Abstract

Right ventricular dysfunction (RVD) parameters are increasingly important features in heart failure with preserved ejection fraction (HFpEF). We sought to evaluate the prognostic impact of a progressive RVD staging system by combining the tricuspid annular plane systolic excursion (TAPSE) to pulmonary artery systolic pressure (TAPSE/PASP) ratio with functional tricuspid regurgitation (TR) severity. We prospectively included 1355 consecutive HFpEF patients discharged for acute heart failure (HF). Of them, in 471 (34.7%) patients, PASP could not be accurately measured, leaving the final sample size to be 884 patients. Patients were categorized as Stage 1: TAPSE/PASP ≥ 0.36 without significant TR; stage 2: TAPSE/PASP ≥ 0.36 with significant TR; stage 3: TAPSE/PASP < 0.36 without significant TR; and stage 4: TAPSE/PASP < 0.36 with significant TR. By the 1 year follow-up, 207 (23.4%) patients had died. We found a significant and graded association between RVD stages and mortality rates (15.8%, 25%, 31.2%, and 45.4% from stage 1 to stage 4, respectively; log-rank test, *p* < 0.001). After multivariable adjustment, and compared to stage 1, stages 3 and 4 were independently associated with mortality risk (HR: 1.8219; 95% CI 1.308–2.538; *p* < 0.001 and HR = 2.2632; 95% CI 1.540–3.325; *p* < 0.001, respectively). A RVD staging system, integrating TAPSE/PASP and TR, provides a comprehensive and widely available tool for risk stratification in HFpEF.

## 1. Introduction

Heart failure with preserved ejection fraction (HFpEF) is becoming the dominant form of heart failure (HF). No evidence-based treatments are available and the morbimortality burden remains high [1,2]. While attention in HFpEF has traditionally focused on changes affecting the left heart [3], recent studies have highlighted that right ventricular (RV) dysfunction (RVD) and pulmonary hypertension (PH) are common features that contribute importantly to the pathophysiology and prognosis of this syndrome [1,3,4,5].

Beyond other potentially involved mechanisms, the development of RV dysfunction is mainly related to elevated pulmonary pressures [3,4]. In HFpEF, the RV is markedly dependent on afterload, and the coupling of RV systolic function for a given pressure overload is commonly impaired, so RV contractility gets worse with progressively higher vascular loading [6,7]. RV–pulmonary artery (PA) coupling has emerged as a global index of RV performance and right length–force relationships [8,9]. The ratio of tricuspid annular plane systolic excursion/systolic pulmonary artery pressure (TAPSE/PASP) measured by echocardiography has been proposed as a noninvasive index of RV–PA coupling, showing excellent correlations with PA compliance and distributing accordingly in the PA compliance and pulmonary vascular relationship by invasive data [10,11]. Functional tricuspid regurgitation (TR) is commonly the final consequence of right-side pressure overload and RV–PA uncoupling, leading to further RV remodeling and dysfunction, perpetuating the process [4,5,12]. All of these RV dysfunction (RVD) parameters have been shown to be strongly related to outcomes in HFpEF in isolation [8,10,13,14,15], but little is known of the prognostic value of the interplay between them. Our aim was to create a comprehensive noninvasive staging system of progressive RVD features in HFpEF, integrating both TAPSE/PASP and TR, and to assess the impact of these stages on survival after a hospitalization for acute HF.

## 2. Experimental Section

### 2.1. Study Group and Protocol 

We prospectively included a consecutive observational cohort of 1355 HFpEF patients who were discharged alive after a hospitalization for acute HF in the cardiology department of a tertiary-care teaching hospital in Valencia (Spain). During index hospitalization, a comprehensive set of demographics, medical history, vital signs, 12-lead electrocardiogram, standard laboratory (72 ± 12 hours after admission), echocardiographic parameters, and treatments at discharge were routinely recorded using pre-established registry questionnaires. Most of the patients exhibited on admission acute decompensated HF (82.7%), followed by acute pulmonary edema (12.6%). Patients were enrolled from 1 January 2007 to 1 August 2016. Patients with new-onset or acutely decompensated HF were enrolled in the registry. HFpEF was defined according to the European Society of Cardiology Clinical Practice Guidelines [2]. We retrospectively evaluate this cohort for the aim of this study. In 471 (34.7%) patients, PASP could not be measured accurately due to the lack of a proper Doppler TR signal, leaving a final sample size of 884 patients. Treatment strategies were individualized following established guidelines that were operating at the time the patient was included in the registry.

### 2.2. Echocardiography

**Figure 1:**
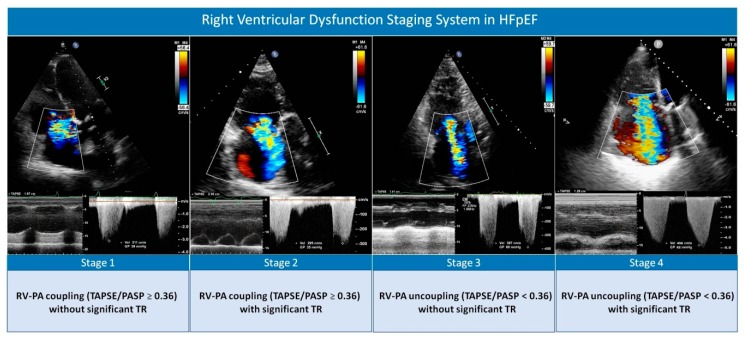
Proposed echocardiographic right heart dysfunction staging classification system based on right ventricular–pulmonary artery coupling and functional tricuspid regurgitation severity. HFpEF: heart failure with preserved ejection fraction; RV–PA: right ventricular–pulmonary artery; TAPSE/PASP: tricuspid annular plane systolic excursion to pulmonary artery systolic pressure ratio; TR: tricuspid regurgitation.

In all patients, a 2-dimensional transthoracic echocardiogram was performed during index hospitalization (96 ± 24 hours after admission) using the left lateral decubitus position. Three commercially available systems were used throughout the study: Agilent Sonos 5500, iE33, and EPIQ 7 (Philips, Massachusetts, USA). Patients were clinically stable and free from intravenous therapies by the time of the examination. All images were recorded with the second harmonic at the time of end-expiration. Left ventricular ejection fraction was assessed by the biplane Simpson method. Left ventricular diastolic dysfunction was evaluated using transmitral flow Doppler velocities and tissue Doppler imaging-derived mitral annulus velocities. PASP was estimated by measuring the maximum continuous Doppler-derived velocity of the TR jet, following established recommendations [16]. Right atrial pressure was estimated in the subcostal view according to inferior vena cava (IVC) size and its breathing-related collapsibility, following a normal sniff as follows: 3 mmHg if IVC < 21 mm that collapses > 50%, 15 mmHg if IVC > 21 mm that collapses < 50%, and an intermediate value of 8 mmHg in the situations in which IVC diameter and collapse did not fit this paradigm [16]. TAPSE was tracked in the four chamber view per M-mode, as recommended [16]. RV–PA coupling was evaluated by calculating the ratio of TAPSE to echocardiographic-derived PASP. This is a noninvasive index of global RV performance against a given degree of afterload, proposed by Guazzi et al. [8], that has been strongly related to outcomes ever since as a noninvasive measure of pulmonary artery distensibility [6,10,11]. Previous studies have shown that the lower the TAPSE/PASP ratio, the worse the outcome, but a prognostic cut-off of <0.36 was identified in most of the studies [8,9,11] and has been acknowledged in a recent statement by the Heart Failure Association of the European Society of Cardiology [4]. So, the variable was categorized according to this cut-off value for statistical analyses. In addition, we assessed the adequacy of this cut-off in our cohort by exploring the functional form of this ratio as a continuous variable in the multivariate model for all-cause mortality. According to current recommendations, the severity of TR was assessed by using an integrated multi-parametric score that includes qualitative and semi-quantitative parameters [17]. Color-flow Doppler was examined in the apical 4-chamber, parasternal short and long axis, and subcostal views. Quantitative measures of the TR jet by proximal isovelocity surface area method were available in less than 10% of patients, so it was not used in this analysis. TR severity grade was assigned by the cardiac sonographer and was confirmed and finally established by another investigator of the study who was blinded to clinical data. The TR severity score comprises four categories: (1) mild, (2) moderate, (3) moderate-to-severe, and (4) severe. All regurgitation jets with a vena contracta ≥ 7 mm and/or systolic flow reversal in hepatic veins were considered to be severe (grade 4). Grades 3 or 4 were labeled as significant TR. RVD stages were created combining both TAPSE/PASP and significant TR, as follows: Stage 1: TAPSE/PASP > 0.36 with no significant TR; stage 2: TAPSE/PASP > 0.36 with significant TR; stage 3: TAPSE/PASP < 0.36 with no significant TR; and stage 4: TAPSE/PASP < 0.36 with significant TR (Figure 1).

### 2.3. Outcomes

The primary endpoint of the study was 1-year all-cause mortality. The survival status was ascertained by reviewing the electronic medical records, corroborated by an investigator who was blinded to the RVD staging system. Death due to cardiovascular (CV) causes and death due to HF were evaluated as secondary outcomes. CV death included sudden death, death due to HF progression, myocardial infarction, stroke, arrhythmia, or thromboembolic disease. Unknown deaths were also considered as CV deaths.

### 2.4. Ethical Concerns

The observational registry was prospectively designed, conformed to the principles outlined in the 1975 Declaration of Helsinki, and approved by the institutional local review ethical committee. All patients gave informed consent.

### 2.5. Statistical Analysis

Continuous variables were expressed as mean ± standard deviation (SD) or median (interquartile range (IQR)), as appropriate. Discrete variables were summarized as percentages. Baseline characteristics were compared among categories with Pearson’s chi-square and *p* values for trend tests for categorical or continuous variables, respectively. The association between the RVD staging system and 1-year all-cause mortality was evaluated using multivariable Cox proportional hazard models, and the results were expressed as hazard ratios (HR) with 95% confidence intervals (CI). Candidate covariates were initially chosen based on previous medical knowledge; then, a backward stepwise selection was performed. The linearity assumption for all continuous variables was simultaneously tested and the variable transformed, if appropriate, with fractional polynomials. The proportionality assumption for the hazard function over time was tested by means of the Schoenfeld residuals. Final multivariate analysis included all the following covariates assessed during index admission: age, gender, systolic blood pressure at admission, heart rate at admission, Charlson comorbidity index, bundle branch block, left atrial size, blood urea nitrogen, and plasma N-terminal pro-B-type natriuretic peptide (NT-proBNP). The detailed multivariate model including all the covariates and their estimates is presented in the Supplementary Materials (Table S1). The mean variance inflation factor of the covariates in the multivariable model was 1.2. For evaluating CV and HF-related mortality, Cox regression models adjusting for non-CV and non-HF-related death as competing events were used, respectively [18]. The discriminative abilities of the models were assessed by Harrell’s C-statistics.

A 2-sided *p* value of <0.05 was considered to be statistically significant for all analyses. All statistical analyses were performed using STATA 14.1 (StataCorp. 2014. Stata Statistical Software: Release 14.1. College Station, TX, USA).

## 3. Results

### 3.1. Study Population Characteristics

**Figure 2:**
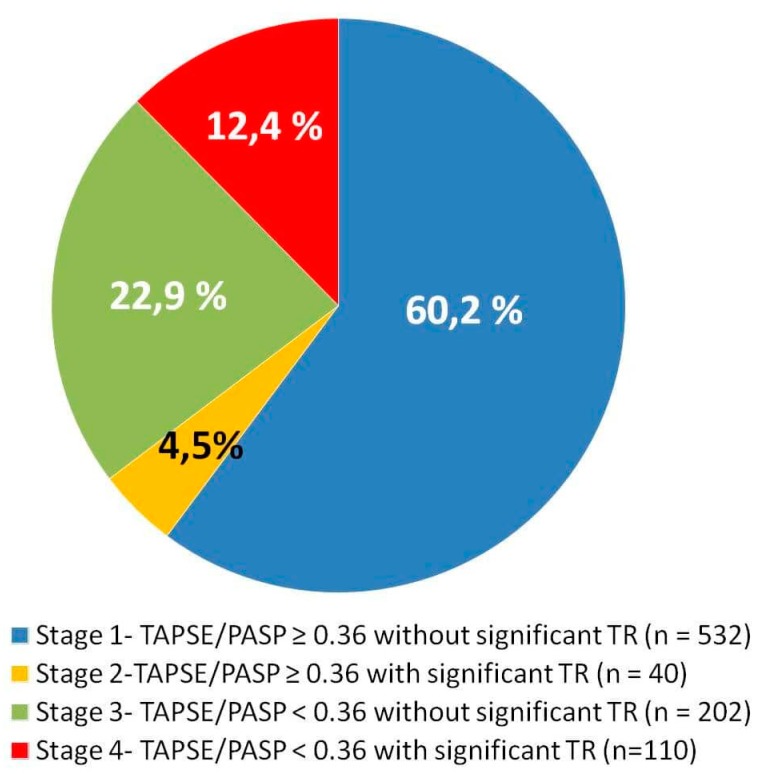
Distribution of right ventricular dysfunction stages in the total study population. TAPSE/PASP: tricuspid annular plane systolic excursion to pulmonary artery systolic pressure ratio; TR: tricuspid regurgitation.

Baseline clinical characteristics for the overall study cohort (mean age 76.4 ± 9.6 years, 67% female) are listed in Table 1. Mean TAPSE, PASP, and TAPSE/PASP were 18.8 ± 3.5 mm, 48.8 ± 20.3 mmHg, and 0.44 ± 0.17, respectively. The proportion of patients with TR grades 1, 2, 3, and 4 were 48.3%, 28.3%, 12.7%, and 4.3%, respectively. The distribution of patients according to the RVD stages were as follows: 532 patients (60.2%) were in stage 1 (TAPSE/PASP ≥ 0.36 and no significant TR); 40 patients (4.5%) were in stage 2 (TAPSE/PASP ≥ 0.36 and significant TR); 202 patients (22.9%) were in stage 3 (TAPSE/PASP < 0.36 and no significant TR); and 110 patients (12.4%) were in stage 4 (TAPSE/PASP < 0.36 and significant TR) (Figure 2).

Table 1 summarizes the baseline characteristics of the study cohort according to RVD stages. Patients with advanced RVD were older, more likely to be female and suffered from a poor functional class prior to admission. Likewise, they showed a higher prevalence of atrial fibrillation, more renal dysfunction, higher NT-proBNP, more surrogates of congestion, greater mitral regurgitation, and more advanced features of diastolic dysfunction.

### 3.2. All-cause Mortality Across RVD Stages

**Figure 3:**
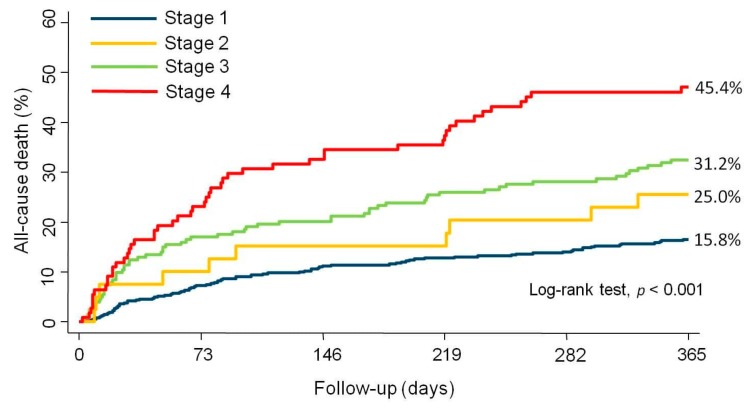
Kaplan–Meier estimates for the cumulative event rates of all-cause mortality according to the stage of right ventricular dysfunction.

At the 1 year follow-up, 207 (23.4%) patients had died. We found a stepped increase in mortality rates when moving from lower to higher RVD stages (15.8%, 25%, 31.2%, and 45.4% from stage 1 to stage 4, respectively (log-rank test, *p* < 0.001)). Kaplan–Meier curves show how trajectories of survival across RVD stages separated early and differences continued increasing along the entire follow-up (Figure 3). It is noteworthy that nearly half of the patients in stage 4 died at the 1 year follow-up.

After multivariable adjustment, and compared to stage 1, stage 3 (TAPSE/PASP < 0.36 without significant TR), and particularly stage 4 (TAPSE/PASP < 0.36 with significant TR) were independently and strongly associated with mortality risk (HR: 1.8219, 95% CI: 1.308–2.538, *p* < 0.001; and HR = 2.2632; 95% CI: 1.540–3.325, *p* < 0.001, respectively). 

**Figure 4:**
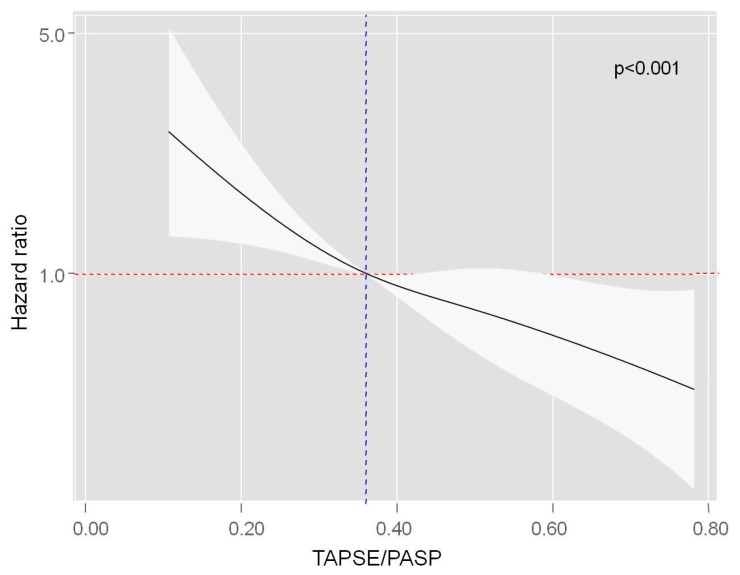
Functional form of TAPSE/PASP ratio evaluated as a continuous variable in the multivariable model for all-cause mortality.

TAPSE/PASP was inversely and almost linearly associated with the risk of all-cause mortality evaluated as a continuous variable. The gradient of risk increased significantly below 0.36, according to the proposed cut-off of the variable (Figure 4). Interestingly, no prognostic differential effect of TAPSE/PASP was found across TR severity, endorsing the combination of both parameters for mortality risk stratification in HFpEF (*p* value for interaction = 0.358, Figure S1).

The estimates of risk for all-cause mortality across different stages in the univariable and multivariable Cox regression analyses are listed in Table 2. Harrell’s C-statistic of the multivariate model was 0.72.

In sensitivity analyses, no significant interactions were found across RVD stages when adjusting for different congestion features such as peripheral edema, pleural effusions, natriuretic peptides, or clinical presentation, reflecting a homogeneous prognostic effect of the proposed staging system across different clinical features (Table S2). Patients in which PASP could not be estimated because of the absence of any discernible TR jet (n = 471) did not significantly show an increased mortality risk when compared to patients in stage 1 (HR = 1.444, 95% CI: 0.947–2.203, *p* = 0.088). When forcing the inclusion of this category in the staging system, results did not substantially change, and patients with both RV–PA uncoupling and significant TR (stage 4) showed the highest mortality risk (Table S3).

### 3.3. CV and HF-Related Mortality Across RVD Stages

**Figure 5:**
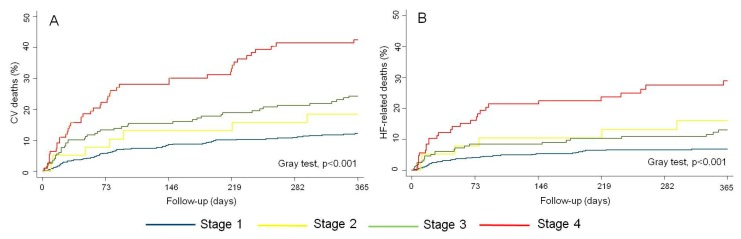
(**A**) Cumulative event rates of cardiovascular mortality risk across right ventricular dysfunction stages. (**B**) Cumulative event rates of heart failure-related mortality across right ventricular dysfunction stages. CV: cardiovascular; HF: heart failure.

At the 1 year follow-up, 157 (17.7%) patients had died from a CV etiology (75.8% of all deaths), and 91 of those (6.7%) were HF-related deaths (43.9% of all deaths). There was a stepwise increase in CV-mortality rates per increasing RVD stages (11.5%, 17.5%, 22.3%, and 40.0%; from stage 1 to stage 4, respectively). Similarly, patients in the most advanced RVD stages also showed the highest rate of HF-related death (6.4%, 15%, 11.4%, and 25.4%, from stage 1 to stage 4, respectively). Cumulative incidence curves showed a marked, progressive, and significant separation of curves along the first months of follow-up for both endpoints, especially for patients in stage 4 (Gray test, *p* < 0,001), as is shown in Figure 5.

After multivariable adjustment, the RVD staging system remained independently associated with the risk of both CV and HF-related mortality. with regard to CV mortality, stage 3 (TAPSE/PASP < 0.36 without significant TR), and stage 4 (TAPSE/PASP 0.36 with significant TR) remained significantly associated with the endpoint when compared to stage 1 (Table 2). For HF-mortality, when compared to the reference category, stage 2 (significant TR with TAPSE/PASP > 0.36) and stage 4 were independently related to the outcome. However, a significant excess of risk was not reached for stage 3 (Table 2). All estimates of risk for all-cause CV and HF-mortality risk are shown in Table 2.

## 4. Discussion

In this cohort of patients with HFpEF with a recent hospitalization for acute HF, a simple and comprehensive RVD echo-derived staging system, integrating TAPSE/PASP and functional TR, provided useful information for 1-year risk stratification. This RVD score showed a positive and graded association with mortality. Of note, about half of the patients in stage 4 (RV–PA uncoupling and significant TR) died during the first year after discharge, and after a thorough adjustment including well-established prognosticators and potential confounders, patients at higher stages exhibited an increased risk of all-cause, CV, and HF-related death. As far as we know, this is the first study to evaluate the additive prognostic value of RV–PA coupling and TR in HFpEF.

Left heart dysfunction has been traditionally considered the landmark of HFpEF, but this paradigm has changed over the years [1,3,4,5]. Currently, HFpEF is recognized as a complex and heterogeneous syndrome in which different cardiac and extracardiac abnormalities play a relevant pathophysiological role, beyond diastolic dysfunction [1,2,5]. Several studies have highlighted that RVD and PH are common features in HFpEF, present in up to 30% and 80% of the patients [4,7,14], respectively, and both of them contribute importantly to the clinical expression and prognosis of the syndrome, as has been recently recognized in a position statement on behalf of the Heart Failure Association of the European Society of Cardiology [4].

Several right heart parameters have been evaluated in recent years in patients with HFpEF [4]. However, some of them are invasively obtained or are still experimental [3,4]. In contrast, noninvasive parameters such as TAPSE, PASP, or TR severity are well-established proxies of right heart dysfunction and are widely available. They all have a strong prognostic value and are closely interrelated. However, their prognostic implications have been often evaluated separately and there is little data of the prognostic value of the interplay between them [4,5,6,7,8,9,10,11,14]. Characterization of HFpEF should ideally integrate metrics of RV and PA function, including tricuspid valve evaluation [3,19]. Given the fact that functional TR is usually the consequence of both RVD and PH, and its presence causally contributes to further RV remodeling, dysfunction, and progression of the disease [12,15], we believe a simple method integrating the continuum of RVD process in HFpEF, by combining metrics of RV, PA function, and tricuspid valve evaluation seems pertinent. All of these parameters should be used in evaluation and risk stratification, but also potentially as therapeutic targets in HFpEF, but ideally they should not be evaluated in isolation.

Many years ago, Guazzi et al. proposed a noninvasive global marker of RVperformance and RV–PA coupling, namely the TAPSE/PASP ratio [8]. This is an index that can be easily obtained by echocardiography and can be taken as an in vivo marker of changes in RV length versus developed forces. Data confirming its correlation with invasive RV Ees/ea in HFpEF is lacking. However, the TAPSE/PASP ratio has been shown to have an excellent correlation with invasive PA compliance, distributing along the exponential relationship between PA compliance and pulmonary circulation resistance [10,11], and showing an optimal correlation with the pre-capillary component of PH in HFpEF [20]. This index is a strong predictor of mortality and rehospitalizations in HFpEF beyond its components in isolation [8,10,11,20,21], and its value has been expanded to other conditions beyond HF, such as arterial PH or severe aortic stenosis [22,23]. However, for a global assessment of the RVD severity, it seems mandatory to evaluate the competency of the tricuspid valve, which should no longer be considered as an epiphenomenon. TR has a central role in RVD. It is the pathophysiological consequence of both RV remodeling and PH, leading to further valve tethering and RV enlargement, resulting in more TR in a vicious cycle. Thus, TR is the consequence of RV–PA uncoupling and maladaptive RV remodeling [12,19,24]. Its clinical and prognostic value in HF should not be neglected. TR causes fluid retention and systemic congestion, resulting in inflammation, neuro-hormonal activation, and multi-organ damage [24]. In HFpEF, TR is a strong predictor of death and rehospitalizations [13,25] and increases as RVD progresses over time [26]. In a recent meta-analysis including >30,000 patients with significant TR in different cardiac scenarios, TR was associated with an increased mortality risk independent of RV failure or PH [27]. On the other hand, when a significant functional TR is present, patients with RV failure (but no RV enlargement) have worse clinical outcomes [28]. This is in line with our data, in which TAPSE/PASP prognostic value is not significantly affected by the severity of TR, whereas significant TR provides additive and complementary prognostic value to the RV–PA coupling index. So, we hypothesize that including TR in a staging model could improve risk stratification beyond TAPSE/PASP in patients with HFpEF. As has been shown in our study, patients with reduced TAPSE/PASP and significant TR showed the highest risk of mortality, representing a very high-risk advanced phenotype.

In line with our proposed staging system, Bax et al. recently proposed a new staging system for evaluating patients with ischemic heart failure [29]. In this stepwise imaging assessment, the most advanced stage belongs to patients with significant TR as a result of RV remodeling and PH, as was proposed in our study. In the setting of aortic stenosis, another echo-derived staging was recently recommended [30,31]. In this scenario, RV failure was considered to be the most advanced stage. We believe that in the natural history of RVD, RV failure and PH usually precede the development of severe functional TR. This situation belongs to the stage 3 of our proposed schema and is not uncommon (23% of our cohort). On the other hand, sometimes the degree of TR can exceed the magnitude of RV failure, but it is quite uncommon that functional TR may be present without structural RVD (4% of patients in our cohort, stage 2). This stage may include patients with structural valve disease that could have been skipped in the cardiac imaging evaluation. The final step of RVD is determined by the development of significant functional TR, reflecting the failure of the RV. Significant TR is a tipping point in the clinical evolution of patients with HFpEF [4,12,19,24]. In our opinion, the ominous prognosis of patients with both RV failure and significant TR in our cohort (stage 4) confirms the adequacy of this proposed staging system in HFpEF.

Left atrial enlargement is an independent risk factor for mortality in our study, beyond RVD features. Left atrial cardiomyopathy is a key mechanism in the pathophysiology and clinical expression of HFpEF [32]. However, at this stage, as a result of RV damage and pulmonary circulation remodeling, RVD becomes the dominant phenotype in many patients, overwhelming left heart dysfunction features. Right-sided dysfunction and failure, rather than left heart disease, seems to become a crucial factor of systemic multi-organ dysfunction and prognosis in HF [33].

In summary, for a global assessment of the RVD severity in HFpEF, it seems mandatory to evaluate the competency of the tricuspid valve, which should no longer be considered as an epiphenomenon. A simple and widely available staging system, including TAPSE/PASP and TR severity, was significantly and strongly associated to 1-year mortality risk. These data support the clinical use of both parameters in combination for risk stratification in HFpEF, and suggest considering right heart features, such as TAPSE/PASP and TR, as potential therapeutic targets in this complex syndrome

### Limitations

First, this is a single-center observational study in which hidden bias might be operating. Second, echocardiographic studies were not reviewed by an independent core laboratory external to the sonographers. Third, TAPSE has caveats as an index of RV systolic function [16]. Nonetheless, it is a widely available parameter recommended in HFpEF given its simplicity and prognostic data [4]. Advanced imaging techniques, such as RV strain, do not seem to increase the prognostic value of the TAPSE/PASP index [34]. Fourth, a different distribution of stages and/or RV parameters might be designed Others parameters such as S’ or fractional area change were available in a small subset of patients (26% of the cohort) and were not evaluated. However, from our point of view, this stepwise assessment was pathophysiologically considered to be the most adequate and simple tool for an RVD staging in HFpEF. Additionally, this staging system may be easily incorporated into clinical daily practice. Fifth, in patients with very severe TR, Doppler PASP estimation may be inaccurate due to an early equalization of RV and right atrial pressures [16]. Beyond the simplicity of this noninvasive index, hemodynamic evaluation should be required for better characterization in selected patients. Finally, the lack of longitudinal assessment of these RVD parameters precluded inferring the influence of their changes over time on prognosis. 

## 5. Conclusions

An RVD staging system, integrating the TAPSE/PASP ratio and functional TR severity, is independently related to a reduced survival in patients with HFpEF. Patients with RV-PA uncoupling and significant TR are a very high risk subgroup. Further studies should confirm these findings and consider RVD stage severity for risk stratification, monitoring, and tailoring treatments in this complex syndrome. 

## Figures and Tables

**Table 1:** Patient characteristics across right ventricular dysfunction stages.

	Total Population (n = 884)	TAPSE/PASP ≥ 0.36 without Significant TR (n = 532)	TAPSE/PASP ≥ 0.36 with Significant TR (n = 40)	TAPSE/PASP < 0.36 without Significant TR (n = 202)	TAPSE/PASP < 0.36 with Significant TR (n = 110)	*p* Value
Age, years	76.1 ± 9.7	75.9 ± 9.9	74.0 ± 11.1	77.7 ± 8.9	77.2 ± 8.3	0.036
Women	569 (64.4)	339 (63.7)	30 (75.0)	144 (71.3)	82 (74.5)	0.041
First HF admission	494 (55.7)	332 (62.4)	23 (57.5)	96 (47.5)	48 (43.6)	<0.001
Prior NYHA class III/IV	159 (17.9)	63 (11.2)	9 (22.5)	55 (27.2)	32 (29.1)	<0.001
Hypertension	700 (79.2)	429 (80.6)	27 (67.5)	163 (80.7)	81 (73.6)	0.097
Diabetes Mellitus	347 (39.2)	208 (39.1)	13 (32.5)	87 (43.1)	39 (35.4)	0.445
Dyslipidemia	452 (51.1)	284 (53.4)	18 (45.0)	100 (49.5)	50 (45.4)	0.346
Current smoker	59 (6.7)	48 (9.0)	4 (10.0)	5 (2.5)	2 (1.8)	0.002
Ischemic heart disease	185 (20.9)	112 (21.0)	7 (17.5)	51 (25.2)	15 (13.6)	0.107
Charlson index >2	274 (31)	160 (30.1)	9 (22.5)	71 (35.1)	34 (30.9)	0.363
Heart rate (beats/min)	96.4 ± 30.7	98.8 ± 31.7	94.7 ± 29.1	94.5 ± 29.2	89.4 ± 27.4	0.005
SBP (mmHg)	144.5 ± 29.6	146.8 ± 29.8	133.0 ± 17.1	145.4 ± 32.3	135.6 ± 24.4	<0.001
DBP (mmHg)	77.7 ± 18.9	79.2 ± 19.5	74.8 ± 15.9	76.7 ± 18.1	73.5 ± 16.8	0.017
QRS >120 ms	202 (22.8)	122 (22.9)	7 (17.5)	43 (21.3)	30 (27.3)	0.541
Atrial fibrillation	547 (61.9)	297 (55.8)	31 (77.5)	139 (68.8)	80 (72.7)	<0.001
Hemoglobin (g/dL)	11.9 ± 1.8	11.9 ± 1.9	11.9 ± 1.8	11.8 ± 1.6	12.0 ± 1.9	0.604
Sodium (mEq/l)	138.2 ± 5.0	138.5 ± 4.7	138.2 ± 4.2	138.0 ± 5.5	137.3 ± 5.7	0.173
NT-proBNP (pg/mL) *	3653 (5628)	3088 (4351)	2026 (3280)	5282 (7017)	5635 (6364)	<0.001
Serum Creatinine (mg/dL)	1.22 ± 0.59	1.19 ± 0.59	1.10 ± 0.53	1.26 ± 0.58	1.31 ± 0.61	0.016
BUN (mg/dL)	60.9 ± 33.1	56.8 ± 29.1	56.5 ± 30.8	65.7 ± 35.7	73.7 ± 41.0	<0.001
GFR ( mL/min/1.73 m^2^)	61.9 ± 29.8	63.2 ± 25.9	65.8 ± 25.3	59.9 ± 30.5	58.3 ± 43.9	<0.001
CA125 (U/mL) *	56 (103)	48 (83)	79 (126)	60 (98)	99 (146)	0.001
LVEF (%)	61.7 ± 7.5	61.8 ± 7.2	62.1 ± 7.6	61.8 ± 8.2	61.2 ± 7.7	0.864
LAD (mm)	45.7 ± 8.2	43.9 ± 7.4	48.3 ± 11.2	46.5 ± 7.3	51.5 ± 9.1	<0.001
LAVI (mL/m^2^)	44.6 ± 12.1	41.3 ± 11.5	43.9 ± 12.9	46.4 ± 15.7	50.0 ± 16.9	<0.001
DT (ms)	222.5 ± 62.4	225.3 ± 59.2	241.4 ± 77.2	214.4 ± 60.1	216 ± 72.0	0.026
E/e’ ratio	19.3 ± 11.3	18.7 ± 11.8	19.3 ± 10.5	21.3 ± 10.6	19.1 ± 9.4	0.359
TAPSE (mm)	19.2 ± 3.3	19.9 ± 3.2	20.6 ± 4.0	16.8 ± 3.0	16.8 ± 2.8	<0.001
S’ tricuspid wave (cm/s)	11.3 ± 3.1	11.8 ± 3.2	12.0 ± 3.0	9.9 ± 2.5	9.9 ± 2.6	<0.001
PASP (mm Hg)	47.5 ± 16.0	38.8 ± 38.4	45.7 ± 9.2	63.7 ± 26.7	70.4 ± 16.4	<0.001
Functional MR III/IV	188 (21.3)	79 (14.8)	14 (35.0)	48 (23.8)	47 (42.7)	<0.001

Data given as n (%), mean ± standard deviation or median (IQR) *. BUN: blood urea nitrogen; CA125: carbohydrate antigen 125; DBP: diastolic blood pressure; DT: deceleration time; GFR: glomerular filtration rate; HF: heart failure; MR: mitral regurgitation; NT-proBNP: amino-terminal pro-brain natriuretic peptide; NYHA: New York Heart Association; LAD: left atrial diameter; LVAI: left atrium volume index; LVEF: left ventricular ejection fraction; PASP: pulmonary arterial systolic pressure; PH: pulmonary hypertension; SBP: systolic blood pressure; TAPSE: tricuspid annular plane systolic excursion; TR: tricuspid regurgitation.

**Table 2:** Univariable and multivariable estimates of risk of the proposed right ventricular dysfunction (RVD) stages in the Cox regression analyses for all-cause, cardiovascular (CV), and heart failure (HF)-related mortality.

	HR (95% CI)	*p* Value	HR (95% CI)	*p* Value
	**All-cause Mortality**			
	**Unadjusted**		**Adjusted** *	
Stage 1 (reference)				
Stage 2	1.634 (0.848–3.149)	0.142	1.482 (0.755–2.912)	0.253
Stage 3	2.209 (1.593–3.062)	<0.001	1.822 (1.308–2.538)	<0.001
Stage 4	3.568 (2.513–5.066)	<0.001	2.263 (1.540–3.325)	<0.001
	**CV mortality**			
	**Unadjusted**		**Adjusted** *	
Stage 1 (reference)				
Stage 2	1.552 (0.711–3.388)	0.270	1.456 (0.640–3.309)	0.640
Stage 3	2.091 (1.423–3.074)	<0.001	1.730 (1.157–2.585)	0.007
Stage 4	4.207 (2.859–6.192)	0.001	2.677 (1.701–4.212)	<0.001
	**HF mortality**			
	**Unadjusted**		**Adjusted** ^†^	
Stage 1 (reference)				
Stage 2	2.403 (1.017–5.676)	0.046	2.565 (1.062–6.193)	0.036
Stage 3	1.843 (1.085–3.130)	0.024	1.546 (0.904–2.644)	0.112
Stage 4	4.438 (2.696–7.305)	<0.001	2.845 (1.562–5.183)	<0.001

Model adjusted for age, gender, systolic blood pressure at admission, heart rate at admission, Charlson comorbidity index, bundle branch block, left atrial size, blood urea nitrogen, and plasma N-terminal pro-B-type natriuretic peptide, and non-CV * and non-HF ^†^ deaths. CI: confidence interval; CV: cardiovascular; HF: heart failure; HR: hazard ratio.

## Supplementary Materials

The following are available online at https://www.mdpi.com/2077-0383/9/3/831/s1, Figure S1. Estimates of risk of TAPSE/PASP evaluated as a continuous variable in patients with or without significant TR in the multivariable model for all-cause mortality, Table S1. All the covariates and their estimates for all-cause mortality in the final multivariable, Table S2. Multivariable model estimates testing the interaction between right ventricular dysfunction stages and different clinical features for all-cause mortality risk prediction: peripheral edema (yes or no), radiological pleural effusions (yes or no), natriuretic peptides (above vs. below median values), and clinical presentation (acute decompensated heart failure vs. other clinical presentations), Table S3. Univariable and multivariable estimates of risk for all-cause mortality including the absence of a tricuspid regurgitation jet as a variable.
