# Supplementary material for: Right Ventricular Dysfunction Staging System for Mortality Risk Stratification in Heart Failure with Preserved Ejection Fraction
Source: J Clin Med. 2020 Mar 18;9(3):831. doi: 10.3390/jcm9030831 (PMC7141269; doi:10.3390/jcm9030831)
Supplement: Supplementary file 1 [file jcm-09-00831-s001.zip › Table S3.docx]

|  | **HR (95% CI)** | **P value** | | **HR (95% CI)** | | **P value** |
| --- | --- | --- | --- | --- | --- | --- |
|  | **All-cause mortality** | | | | | |
|  | **Unadjusted** | | | **Adjusted*** | | |
| Stage 1 (reference) |  | | | | | |
| No TR | 1.444  (0.947-2.203) | | 0.088 | 1.518  (0.978-2.233) | 0.065 | |
| Stage 2 | 2.421  (1.016-5.766) | | 0.046 | 2.449  (1.007-5.947) | 0.048 | |
| Stage 3 | 1.959  (1.154-3.327) | | 0.013 | 1.579  (0.927-2.692) | 0.093 | |
| Stage 4 | 4.742  (2.874-7.823) | | <0.001 | 2.885  (1.690-4.927) | <0.001 | |
