# Supplementary material for: Right Ventricular Dysfunction Staging System for Mortality Risk Stratification in Heart Failure with Preserved Ejection Fraction
Source: J Clin Med. 2020 Mar 18;9(3):831. doi: 10.3390/jcm9030831 (PMC7141269; doi:10.3390/jcm9030831)
Supplement: Supplementary file 1 [file jcm-09-00831-s001.zip › Table S1.docx]

|  | HR (95% CI) | p value |
| --- | --- | --- |
| Age | 1.0410 (1.021-1.061) | <0.001 |
| Male sex | 0.9766 (0.714-1.336) | 0.882 |
| HR at admission | 3.0073 (1.184-7.638) | 0.021 |
| CCI | 0.9827 (0.822-1.175) | 0.848 |
| SBP at admission | 0.9952 (0.990-1.000) | 0.057 |
| BUN  (gr/dl) | 1.0081 (1.004 - 1.012) | <0.001 |
| Bundle branch block | 1.3449 (0.983-1.839) | 0.064 |
| NT-proBNP (pg/ml) | 1.0000 (1.000-1.001) | 0.043 |
| Left atrial size (mm) | 1.0237 (1.007-1.040) | 0.005 |
