# Supplementary material for: Right Ventricular Dysfunction Staging System for Mortality Risk Stratification in Heart Failure with Preserved Ejection Fraction
Source: J Clin Med. 2020 Mar 18;9(3):831. doi: 10.3390/jcm9030831 (PMC7141269; doi:10.3390/jcm9030831)
Supplement: Supplementary file 1 [file jcm-09-00831-s001.zip › Table S2.docx]

|  | | **HR (95% CI)** | **P value** | | **HR (95% CI)** | | **P value** | **P value for interaction** |
| --- | --- | --- | --- | --- | --- | --- | --- | --- |
| **No** **peripheral edema** | | | | | **Peripheral edema** | |  |  |
| Stage 1  (reference) | | | | |  | |  | 0.983 |
| Stage 2 | 1.6278  (0.3818-6.227) | | | 0.511 | 1.4739  (0.6907-3.1449) | 0.316 | |  |
| Stage 3 | 1.7043  (0.8891-3.2932) | | | 0.113 | 1.9138  (1.3047-2.8058) | 0.001 | |  |
| Stage 4 | 2.0624  (0.9070-4.6869) | | | 0.0804 | 2.3405  (1.5232-3.5965) | <0.001 | |  |
|  | **No pleural effusion** | | |  | **Pleural effusion** |  | |  |
| Stage 1  (reference) |  | | |  |  |  | | 0.722 |
| Stage 2 | 1.4569  (0.5717-3.7127) | | | 0.430 | 1.5597  (0.5990-4.0609) | 0.363 | |  |
| Stage 3 | 1.5541  (0.9615-2.5118) | | | 0.072 | 2.2086  (1.3971-3.4941) | 0.001 | |  |
| Stage 4 | 1.9244  (1.0632-3.4831) | | | 0.031 | 2.6139  (1.6087-4.2471) | <0.001 | |  |
|  | **No ADHF** | | |  | **ADHF** |  | |  |
| Stage 1  (reference) |  | | |  |  |  | | 0.3905 |
| Stage 2 | 1.4651  (0.3346-6.4147) | | | 0.612 | 1.5394  (0.7252-3.2675) | 0.261 | |  |
| Stage 3 | 1.0050  (0.4513-2.2380) | | | 0.990 | 2.1438  (1.4868-3.0911) | <0.001 | |  |
| Stage 4 | 2.1139  (0.8918-5.0107) | | | 0.089 | 2.3823  (1.5690-3.6171) | <0.001 | |  |
|  | **NT-proBNP below median** | | |  | **NT-proBNP**  **above median** |  | |  |
| Stage 1  (reference) |  | | |  |  |  | | 0.6045 |
| Stage 2 | 1.2315  (0.4317-3.5176) | | | 0.697 | 1.7046  (0.7176-4.0493) | 0.227 | |  |
| Stage 3 | 1.3194  (0.7078-2.4592) | | | 0.383 | 2.0987  (1.4052-3.1343) | <0.001 | |  |
| Stage 4 | 2.3219  (1.1159-4.8307) | | | 0.024 | 2.2495  (1.4456-3.5004) | <0.001 | |  |
