# Supplementary figures and images for: Right Ventricular Dysfunction Staging System for Mortality Risk Stratification in Heart Failure with Preserved Ejection Fraction
Source: J Clin Med. 2020 Mar 18;9(3):831. doi: 10.3390/jcm9030831 (PMC7141269; doi:10.3390/jcm9030831)

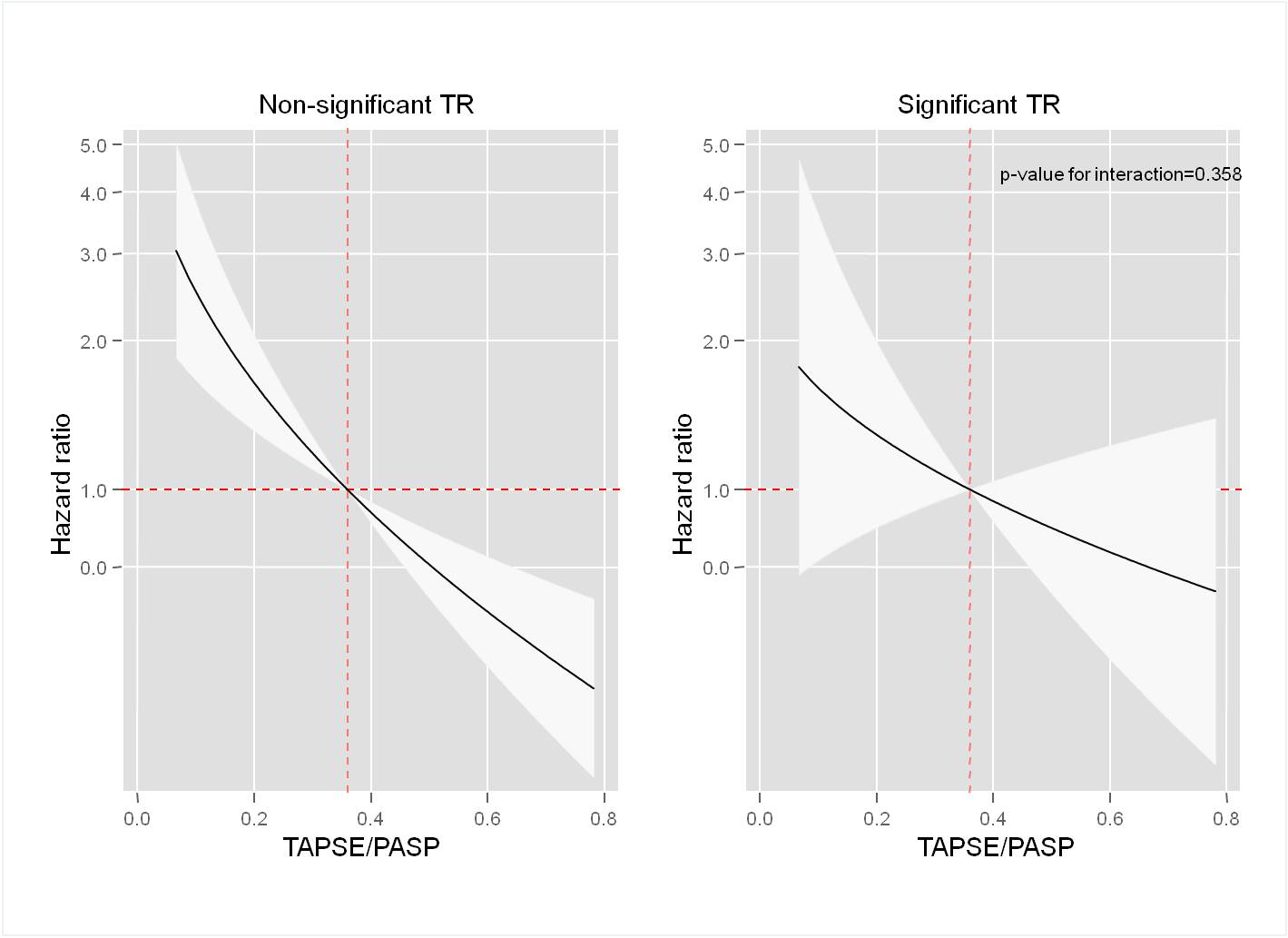

Supplement: Supplementary file 1 [file jcm-09-00831-s001.zip › Figure S1.tiff]
